# Phytoprogestins: Unexplored Food Compounds with Potential Preventive and Therapeutic Effects in Female Diseases

**DOI:** 10.3390/nu13124326

**Published:** 2021-11-30

**Authors:** Stefania Greco, Pamela Pellegrino, Alessandro Zannotti, Giovanni Delli Carpini, Andrea Ciavattini, Fernando M. Reis, Pasquapina Ciarmela

**Affiliations:** 1 Department of Experimental and Clinical Medicine, Università Politecnica delle Marche, 60126 Ancona, Italy; s.greco@staff.univpm.it (S.G.); p.pellegrino@pm.univpm.it (P.P.); a.zannotti@pm.univpm.it (A.Z.); 2 Department of Specialist and Odontostomatological Clinical Sciences, Università Politecnica delle Marche, 60126 Ancona, Italy; g.dellicarpini@staff.univpm.it (G.D.C.); a.ciavattini@univpm.it (A.C.); 3 Department of Obstetrics and Gynecology, Universidade Federal de Minas Gerais, Belo Horizonte 30130-100, Brazil; fmreis@ufmg.br

**Keywords:** female disease, progesterone, phytoprogestins, phytochemical compounds

## Abstract

In recent years, there has been an increasing interest in natural therapies to prevent or treat female diseases. In particular, many studies have focused on searching natural compounds with less side effects than standard hormonal therapies. While phytoestrogen-based therapies have been extensively studied, treatments with phytoprogestins reported in the literature are very rare. In this review, we focused on compounds of natural origin, which have progestin effects and that could be good candidates for preventing and treating female diseases. We identified the following phytoprogestins: kaempferol, apigenin, luteolin, and naringenin. In vitro studies showed promising results such as the antitumoral effects of kaempferol, apigenin and luteolin, and the anti-fibrotic effects of naringenin. Although limited data are available, it seems that phytoprogestins could be a promising tool for preventing and treating hormone-dependent diseases.

## 1. Introduction

In recent years, there has been an increasing interest in alternative and natural methods for the prevention or treatment of female diseases. In particular, many studies have focused on searching for adequate compounds with less side effects than standard hormonal therapies. Although the etiopathogenetic mechanisms of many gynecological diseases, such as endometriosis [1] and uterine fibroids [2] are still not clear, the role of steroid hormones is undoubted. Indeed, there is an important hormonal imbalance, for example, in endometriosis [3], uterine leiomyomas [4], ovarian cancer [5], and breast cancer [6].

The father of medicine, Hippocrates, proclaimed “Let food be the medicine and medicine be the food” around 25 centuries ago. In recent studies, there is a high interest in dietary phytochemicals. Phytochemicals are chemical compounds of natural origin that can be used as therapeutic or preventive agents.

Nutraceutical compounds can exert their effects on health in different ways, including through hormonal activity. Their mechanism of action is: 1. Competition with the hormone for binding to the corresponding receptor, thanks to a structural similarity; 2. Influence on the activity of key enzymes of the biosynthetic pathway, such as in the case of isoflavones, which are moderate aromatase inhibitors, thus reducing estrogen synthesis; 3. Influence on the epigenome by affecting DNA methylation activity, histone modification, and microRNA regulation [7].

Phytoestrogens and phytoprogestins are phytochemical compounds of natural origin, which have estrogenic and progestagenic effects, respectively [8,9]. While phytoestrogen-based therapies have been extensively studied in the clinical setting, treatments with phytoprogestin are still in the preclinical stage, and their potential remains unexplored [8]. Therefore, we decided to review the current evidence supporting the preventive and therapeutic effects of phytoprogestins in female diseases.

## 2. Methods

In this narrative review, we performed a bibliographic search of studies evaluating the effects of dietary phytoprogestins on reproductive cells and tissues and the possible association of these nutritional compounds with gynecological diseases. The search was carried out on Pubmed using combinations of the following terms: phytochemicals [MeSH], flavonoids [MeSH], kaempferol, apigenin, naringenin, luteolin, women, uterine fibroids, endometriosis, ovarian cancer, and breast cancer. The search was narrowed to studies in humans or relevant animal models of human diseases and complemented by screening the reference lists of the selected articles. We also briefly review the pharmacological mechanisms of progesterone receptor activation and progesterone-based therapies in order to provide a background to the discussion of phytoprogestins.

## 3. Progesterone

**Figure 1:**
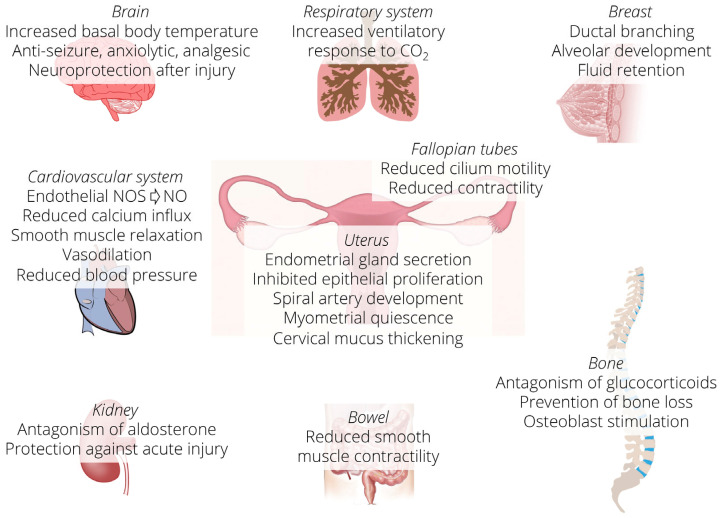
Schematic representation of the organs in which progesterone performs functions. Progesterone acts in reproductive as well as in non-reproductive tissues. NOS = nitric oxide synthase, NO = nitric oxide.

Progesterone is a sex steroid hormone essential in female reproduction, including the menstrual cycle and the establishment and maintenance of pregnancy [10]. The etymology of the name derives from the Latin “pro gestationem” [11], as it allows the endometrium to pass from the proliferative to the secretory stage, facilitating the nesting of the blastocyst and is essential for maintaining pregnancy; in fact, it promotes the uterine growth and suppresses the contractility of the muscular tissue of the uterus (myometrium). In the mammary gland, it promotes the development of the gland for the secretion of milk. In addition, progesterone plays an essential role in the physiology of non-reproductive tissues, such as the cardiovascular system, the central nervous system, and bone tissue. In the brain, progesterone is neuroprotective, and its metabolite allopregnanolone is a GABAergic agonist [12,13] (Figure 1).

Steroids are ancestral molecules [11] characterized by a common base structure of cyclopentane–perhydro–phenanthrene, a polycyclic complex of 17 carbon atoms making a four-ring system. Based on the number of carbon atoms, sex steroids can be categorized into three groups: progesterone and progestins, with 21 carbon atoms, androgens, which have 19 carbon atoms, and finally estrogens, with 18 carbon atoms.

The biosynthesis of steroid hormones is the same in all organs where they are produced, such as the ovary, testis, adrenal cortex, brain, and placenta. The gonadal progesterone is mainly transported by blood to reach the target cells, while the progesterone produced by adrenal gland is mostly locally converted into glucocorticoids and androgens [14]. Progesterone circulates in the bloodstream bound to cortisol-binding globulin (approximately 10%) and serum albumin and has a relatively short half-life of only five minutes. The metabolites mainly produced in the liver are sulfates and glucuronides, which are excreted in the urine. Circulating progesterone is converted by the kidney into a mineralocorticoid, deoxycorticosterone (DOC). During the luteal phase, pregnancy, and administration of exogenous progesterone, most circulating DOC arises from this pathway and may bring unbearable side effects [14].

Progesterone exerts its physiological effect by binding to target cells via specific nuclear progesterone receptors (PR) or by binding to membrane receptors (progesterone receptor membrane component, PGRMC, or mPR). The binding with the nuclear receptors gives rise to a genomic pathway that requires a much longer response than the non-genomic one, which is triggered when progesterone binds to membrane receptors.

**Figure 2:**
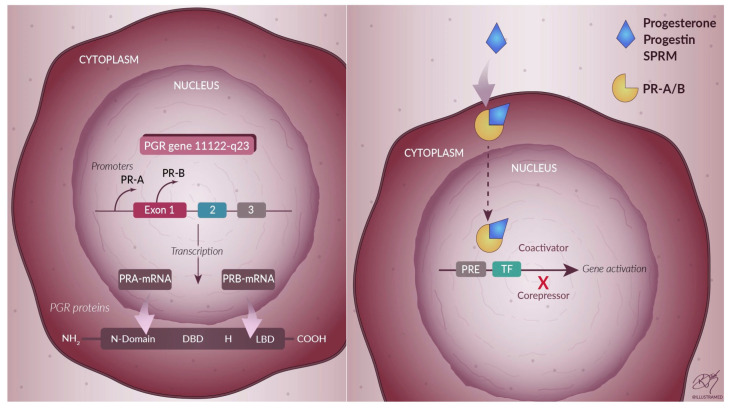
Progesterone receptors and their activation. The nuclear receptor is formed by two promoter regions on the PR gene, one for PRA and one for PRB, and these two promoters allow the synthesis of the two separate mRNA transcripts that code for the two different isoforms PRA and PRB. DBD = DNA-binding domain, H = hinge, LBD = ligand-binding domain, SPRM = selective progesterone receptor modulator, PRE = progesterone responsive element, TF = transcription factor.

PRs are expressed in the human ovary [15], uterus [16], testis [17], brain [18], pancreas [19], bone tissue [20], mammary gland [21] and urinary tract [22]. PRs, together with the receptors for estradiol, mineralocorticoids, glucocorticoids, and androgens, belong to the superfamily of nuclear receptors. The nuclear progesterone receptor consists of a central binding domain for DNA (DBD) and a carboxylic terminal binding domain for the ligand (LBD). In addition, the receptor has transcription activation function (TAF) domains that interact with coactivators and corepressors to regulate the downstream target genes [23] (Figure 2). The newly transcribed progesterone receptor is assembled into an inactive multiprotein chaperone complex in the cytoplasm [24]. The receptor at this level must be inactive [25] since its activation occurs only in the presence of a link with the hormone, which induces a conformational change of the receptor [26].

Two isoforms of PR are transcribed from a single gene by alternative splicing from two distinct promoters [27,28], giving rise to transcripts that encode, respectively, for the protein isoforms A (PRA) and B (PRB) (Figure 2). PRA and PRB are identical in sequence, except that PRA lacks 164 amino acids at the N-terminal, making it the shorter of the two proteins [28].

Progesterone may act through a genomic (slow process) or a non-genomic (fast process) pathway. The classical pathways of progesterone actions are mediated via nuclear receptors. Progesterone enters the cell and binds PRs, inducing their conformational change and dimerization. The complex of progesterone with PR translocates to the nucleus and interacts with DNA-binding elements in the genome, activating the transcription of progesterone-responsive genes (Figure 2)**.** The non-genomic (also called non-classical or extranuclear) progesterone action initiates at the cell surface with the activation of the cytoplasmic PRs or membrane-bound PRs (mPRs) and determines an intracellular signaling that elicits a rapid response [29]. These proteins include the progesterone receptor membrane component 1 (PGRMC1), its counterpart PGRMC2, and the family of membrane progesterone receptors (mPR), also known as PAQR (progestin and adipoQ) receptors [30].

Studies in mice have shown that the elimination of the PRB isoform resulted in the unhealthy development of the mammary gland [31], while the elimination of PRA caused an abnormal development of the uterus and impaired its reproductive function [32]. Therefore, in animals, a dominant expression of one of the two isoforms seems to be necessary for the normal functioning and development of some organs. On the other hand, in humans, all healthy tissues, including those of the mammary gland and uterus, have epithelial cells that express PR with the co-expression of both the PRA and PRB isoforms [33,34]. This condition suggests that the colocalization and thus the cooperative activity of PRA and PRB mediate the action of PR in humans. Although the two isoforms are expressed in the same way in most human tissues, there is a different expression in the endometrium. In fact, during the secretory phase of the menstrual cycle, when there are high levels of circulating progesterone, the PRA isoform is poorly expressed, resulting in a clear predominance of PRB [33].

In breast and endometrial cancers, there are substantial differences in progesterone levels and its isoforms compared to normal tissues. In fact, in healthy tissues deriving from the mammary gland, epithelial cells equally express both PR isoforms [34], while in neoplastic biopsies, it is possible to see a significant increase in the expression, alternatively, of PRA or PRB [34,35]. Similarly, in endometrial cancer, it is common to find only one of the two isoforms expressed, either PRA or PRB, suggesting that the lack of co-expression of both isoforms is an early event of the onset of endometrial cancer [36].

A third isoform (PRC) has been identified in the human placenta [37]. PRC is an isoform with a truncated N-terminal domain, with a molecular mass of approximately 60 kDa, present in the cytoplasm. PRC lacks the first zinc finger of the DBD, but it can still bind progesterone. The actions of PRC are not clear, but it can form heterodimers with PRA and PRB and, in this way, regulate the transcriptional activity of the PR isoforms [37,38].

## 4. Progesterone-Based Drug Therapy

Progestogens are the most common compounds used as drug therapy for the treatment of women’s diseases. Many gynecological diseases are treated with synthetic progestin-based drugs. In the United States, endometrial cancer is one of the most common gynecological cancers, with 46,470 new cases and 8,120 deaths in 2011 [39]. Even if the molecular mechanisms involved in endometrial carcinogenesis are not clear, it seems that chronic exposure to estrogen and its metabolites without sufficient counterbalance of progesterone has proliferative effects [40,41] and is harmful to DNA [42,43]. Based on the antiestrogenic role of progesterone, many patients affected by endometrial cancer may have an indication to progesterone-based therapy, particularly in case of contraindications to surgery or desire for fertility maintenance. Indeed, women with endometrial hyperplasia and well-differentiated endometrial adenocarcinoma show a good response to progestogen therapy [44]. However, as the severity of the disease increases, the efficacy of progestogens decreases [45].

Other estrogen-dependent female pathologies with a high social impact, such as endometriosis, are often treated with progestin therapies [46,47], including synthetic progestins such as medroxyprogesterone acetate or dienogest [48,49]. Uterine fibroids may also be treated with progestins. Since the first reports of decades ago [50], studies have focused on the effects of different progestins on uterine fibroids, with different drug dosages and regimens. For example, medroxyprogesterone acetate [51] and dienogest [52,53] have shown a regressive effect on uterine fibroids.

Moreover, progestogens are widely used as a contraceptive method and in menopausal hormone therapy, in combination with estrogens. These therapies may also have an effect of prevention of ovarian cancer, but they increase the risk of venous thromboembolism and present side effects [54,55]. Therefore, the identification of alternative progestogens is clinically significant. Numerous studies in the literature indicate a great interest in developing phytoprogestogens, such as botanical extracts or food supplements, hoping to provide the beneficial effects of progestins while avoiding the side effects.

Selective progesterone receptor modulator (SPRM) is a class of synthetic ligands that act on the PR and tend to be more tissue-specific than progestins. The mechanism of action of SPRMs occurs through binding to PR, resulting in a conformational change of the receptor. The action can be agonistic, antagonistic, or mixed. The agonist action of SPRMs involves the recruitment of different coactivators to induce transcriptional activity and occurs in tissues where high levels of coactivators are present, while antagonist activity occurs where corepressors are in excess (Figure 2).

When the PR is inactive, SPRMs bind to the receptor and activate it. The binding involves nuclear import, which gives the receptor the property of dimerization. In the nucleus, the dimer interacts with the response element in the DNA, causing the up-regulation or down-regulation of the gene [56,57]. The action of SPRMs also depends on the ratio of PR-A and PR-B in the tissue and on the specific binding affinity of the SPRMs for each receptor isoform [58]. SPRMs have been developed for clinical applications, considering their tissue selectivity and low rate of side effects [59]. Their application is principally for the treatment of uterine fibroids [60], endometriosis [61], and breast cancer [62].

Despite having beneficial effects, for example, in the treatment of uterine fibroids, the prolonged use of SPRMs may lead to endometrial hyperplasia and other side effects. Indeed, it has been shown that long-term use of the SPRM asoprisnil results in long-term damage to the endometrium. Ulipristal acetate has been approved in Canada and Europe as a presurgical therapy for patients with uterine fibroids to control bleeding, and in the United States for emergency contraception. However, it has raised concerns due to liver toxicity [63], as well as telapristone acetate, which was stopped in 2009. Vilaprisan is still under study, and its possible collateral effects are not yet known [64].

## 5. Phytoprogestins

**Figure 3:**
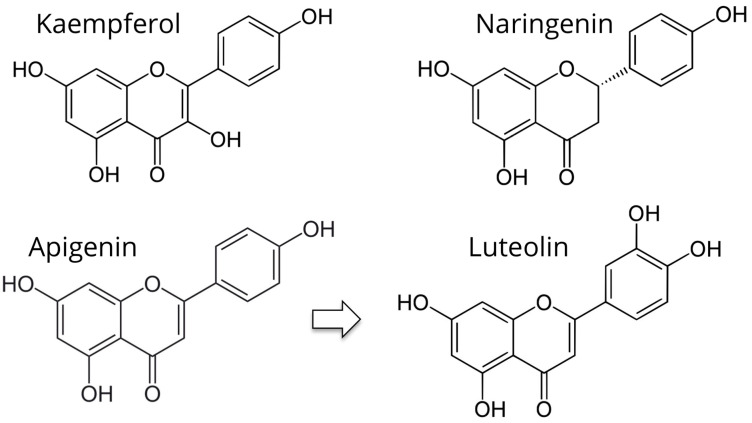
Chemical structure of phytoprogestins.

Phytoprogestins are chemical compounds of vegetal origin that have progesterone-like activity and can function as non-steroidal SPRMs. Unlike estrogenic counterparts, which have been extensively studied, the literature reports much fewer studies on phytoprogestins. The following phytoprogestins have been identified: kaempferol, apigenin, luteolin, and naringenin (Figure 3).

### 5.1. Kaempferol

Kaempferol (KP: 3,5,7-trihydroxy-2-(4-hydroxyphenyl)-4H-1-benzopyran-4-one) is a flavonoid found in several botanical families, including Pteridophyta, Pinophyta, and Magnoliophyta (Figure 3). Flavonoids are a group of secondary metabolites widespread in nature. These substances are known for the benefits of their consumption, which seems to reduce the risk of cancer and cardiovascular diseases [65]. A case–control study showed a 40% reduction (adjusted odds ratio 0.60) in breast cancer risk in Chinese women in the upper quartile of serum KP levels [66]. The risk of epithelial ovarian cancer was also decreased by 40% among women in the highest quintile of KP dietary intake of a large prospective cohort in the USA, the Nurses’ Health Study [67]. Several studies have shown that KP has excellent antioxidant properties. In fact, it is able to decrease, even at low concentrations, the levels of the hydroxyl radical and peroxynitrite, highly reactive species capable of causing severe damage to DNA, proteins, and lipids [68]. In addition, KP has anti-inflammatory properties not only in vitro but also in vivo [69,70].

KP inhibits estrogen receptor-α, causing antiestrogenic effects, depending on the concentration of endogenous estrogens. The antiestrogenic activity of KP results in the inhibition of the growth of hormone-dependent tumors; this activity has been demonstrated in numerous in vitro studies, for example, in endometrial carcinoma cells [71] and two lines of breast cancer cells [72].

In uterine fibroids, despite being hormone-dependent tumors with severe symptoms, the effects of KP have not been extensively studied. KP treatment reduces the expression of the estrogen receptor, thus inhibiting the cell proliferation of human uterine leiomyoma cells in vitro [73], although its therapeutic effect in vivo remains unknown (Table 1).

### 5.2. Apigenin and Luteolin

Apigenin (4’,5,7-trihydroxyflavone) is found in a wide range of plants, including chamomile (*Matricaria recutita*). The traditional use of chamomile as a treatment for insomnia and anxiety has led to investigations of its active constituents, including apigenin. Apigenin is mainly present as a glycosylated compound in significant quantities in vegetables (parsley, celery, onions), fruit (oranges), herbs (chamomile, thyme, oregano, basil), and vegetable drinks (tea, beer, and wine) [80]. Apigenin is considered a phytoestrogen, although it has a much lower potency than other phytoestrogens such as genistein [81]. However, in recent studies, it has emerged that apigenin is also a phytoprogestin. A study found that apigenin reduces the risk of breast tumors in women exposed to prolonged treatment with medroxyprogesterone acetate [82,83]. A study by Horwitz and Sartorius showed that prolonged progestogen therapy could lead to the development of breast cancer through the activation of stem cells that differentiate into cancer cells [84]. In animals subjected to medroxyprogesterone therapy, apigenin administration decreased the incidence of tumors by 50% [82].

Apigenin has an antitumor effect by acting through a variety of mechanisms, including the induction of cell cycle arrest and apoptosis [74], attenuation of phosphorylation of MAP kinase [85] and inhibition of the proinflammatory cytokine interleukin-6 [86]. In vitro studies have shown that treating human breast cancer cell lines [75] with apigenin significantly reduced the expression of vascular endothelial growth factor (VEGF) and its receptor VEGFR-2 [87]. The significant reduction in VEGF disadvantages the tumor growth and development in breast tissue.

Apigenin taken orally is detectable in peripheral blood at concentrations sufficient to be biologically effective [88]. Immediately after ingestion, its concentration increases, and it remains in circulation for a long time, suggesting that it can accumulate within tissues to levels sufficient to exert chemo-preventive effects [89]. Furthermore, apigenin increased the endometrial expression of Hand2, which is a transcription factor stimulated by progesterone. The activation of Hand2 by progesterone allows an antiproliferative action in the endometrium, further suggesting that apigenin is a phytoprogestin. Apigenin appears to be non-toxic even at high doses, as suggested by a study in which it was repeatedly administered to animals up to 50 mg/kg per 10–13 days, and no signs of toxicity were observed. Apigenin seems to reduce endometrial (Ishikawa) cell proliferation regardless of progesterone [90]. In vivo, apigenin is rapidly metabolized to luteolin.

Luteolin, a flavonoid found in more than 300 plant species, many of which are readily available in the human diet, has been demonstrated to be an excellent progesterone antagonist [91] (Figure 3). A study showed that luteolin effectively inhibits the growth of progestogen-dependent human xenograft tumors, inhibiting angiogenesis and limiting the conversion of breast cancer cells to stem cell-like cells [76,77]. Interestingly, preliminary results suggest that luteolin may inhibit the growth of endometriotic lesions in a mouse model [92].

### 5.3. Naringenin

Naringenin (4,5,7-dihydroxy-2-(4-hydroxyphenyl)-2,3-dihydrochromen- 4-one) belongs to the subclass of flavanones (Figure 3). It is a colorless compound that gives the typical bitter taste in citrus, including grapefruit, orange, and lemon [93].

Naringenin has antioxidant, immunomodulatory, anti-inflammatory, nephroprotective, hepatoprotective, neuroprotective, antidiabetic, antitumor, and anti-atherosclerotic properties. In addition, naringenin has a high bioavailability [94,95].

Naringenin is able to inhibit the recruitment and generation of reactive oxygen species (ROS), thereby reducing oxidative stress [78,96]. Moreover, it acts directly on the NF-KB pathway in vitro and in vivo [97]. This signaling pathway is known to be activated by external agents such as pathogens. In the presence of external agents, pro-inflammatory cytokines such as IL-1 and TNF-α are recalled [98]. This stimulation and this recall involve the activation of the IKB kinase complex (IKK), which eventually phosphorylates IKK β. The phosphorylated IKB allows NF-KB to translocate into the nucleus, causing inflammatory responses [99]. Naringenin can prevent the degradation of IKB, inhibiting the transcription activity of NF-KB [98].

In numerous studies, it has emerged that naringenin is also an excellent anti-fibrotic agent [79]. In fact, naringenin was able to decrease the expression of collagen, fibronectin, and Smad3 induced by TGF-β and to inhibit Plasminogen Activator-1 (PAI-1) in hepatic cells [100]. Some of these mechanisms are similar to those fueled by progesterone in uterine fibroids [4].

In a study by Rosenberg et al. [101] it emerged that naringenin may also have progestin-like activity. More specifically, the study showed that the progestin activity of naringenin is weak and acts at concentrations around 10^−5^–10^−6^ M. These concentration levels are similar to those deemed necessary for the action of resveratrol as a weak estrogen [102], but not for the activity of synthetic progestins such as norgestrel and norgestimate. In fact, the biological activity of naringenin compared to norgestimate is about 104-fold lower.

The effects that naringenin as a phytoprogestin could have on diseases such as endometriosis and uterine fibroids remain to be investigated. An in vitro study found that naringenin induced apoptosis and inhibited the proliferation of immortalized cell lines derived from the endocervical epithelium of a premenopausal woman undergoing hysterectomy for endometriosis [103].

## 6. Conclusions

There is large unexplored potential in using plant-derived substances to treat human diseases. Some of these phytochemicals have been characterized as phytoprogestins, based on their similarity with progesterone and their pharmacological interaction with PR, functioning as agonists, partial agonists, or antagonists. At least four phytoprogestins have been studied in vitro with promising results such as the antitumoral effects of KP, apigenin, and luteolin, and the anti-fibrotic effects of naringenin. Although there are limited data in the literature, it appears that phytoprogestins could be a good tool for preventing and treating hormone-dependent diseases such as endometriosis, uterine fibroids, ovarian cancer, and breast cancer, with potential reduction in the side effects of currently available hormone treatments. The next step is to proceed with tests in well-characterized animal models to define the therapeutic mechanisms and safety of these substances, along with observational human studies correlating the dietary ingestion of phytoprogestins with the prevalence and incidence of gynecologic diseases.

## Figures and Tables

**Table 1:** Effects of phytoprogestins that suggest their potential to treat women’s diseases.

Substance	Study Design	Effects	Significance	References
Kaempferol	Experiments in mice and rats	Anti-inflammatory	Could be useful to treat chronic pelvic pain and its causes	[69,70]
	In vitro culture of human neutrophils	Antioxidant	Another potential therapeutic mechanism to treat endometriosis	[68]
	In vitro culture of endometrial cancer cells	Growth inhibition and apoptosis	Could be effective against endometrial hyperplasia and cancer	[71]
Apigenin	In vitro culture of human cancer cell lines	Growth inhibition and apoptosis VEGF inhibition	Could be effective against endometrial hyperplasia and cancer	[74,75]
Luteolin	Human breast tumor xenografts in nude mice	Inhibition of tumor growth and angiogenesis	Could be an adjuvant therapy of breast cancer	[76,77]
Naringenin	Mouse model in vivo	Analgesic, anti-inflammatory and antioxidant	Could be useful to treat chronic pelvic pain and its causes	[78]
	Rat model of hepatic injury in vivo	Antifibrotic	Could be effective to treat uterine fibroids	[79]

## Data Availability

Not applicable.
